# Stroke-like manifestation of a spontaneous spinal epidural hematoma with spontaneous resolution: a case report

**DOI:** 10.1186/s12245-023-00478-0

**Published:** 2023-02-20

**Authors:** Xue Bi, May Na, Swee Min, Kathleen Khoo

**Affiliations:** Emergency Department, Khoo Teck Puat Hospital, National Healthcare Group, 90 Yishun Central, Singapore, 768828 Singapore

**Keywords:** Hematoma, Epidural, Spinal, Stroke, Spine, Paresis, Emergency Service, Hospital

## Abstract

**Background:**

This case report presents the case of a man with no known coagulopathy or preceding trauma, who spontaneously developed a spinal epidural hematoma (SEH). This is an uncommon condition which can have variable presentations including hemiparesis mimicking stroke, resulting in the potential for misdiagnosis and inappropriate treatment.

**Case presentation:**

A 28-year-old Chinese male with no past medical history presented with sudden onset neck pain associated with bilateral upper limbs and right lower limb subjective numbness but intact motor function. He was discharged after adequate pain relief but re-attended the emergency department with right hemiparesis. A magnetic resonance imaging of his spine revealed an acute cervical spinal epidural hematoma at C5 and C6. While admitted, he had spontaneous improvement of his neurological function and was eventually managed conservatively.

**Conclusions:**

SEH, although uncommon, can be a mimic of stroke and it is important to avoid misdiagnosis as it is a time critical diagnosis, and administration of thrombolysis or antiplatelets can lead to unfavourable outcomes. Having a high clinical suspicion can help to guide us in the choice of imaging and interpretation of subtle signs to reach the correct diagnosis in a timely manner. Further research is required to better understand the factors that would favour a conservative approach as opposed to surgical treatment.

## Background

This case reports presents the case of a man with no known coagulopathy or preceding trauma, who spontaneously developed a spinal epidural hematoma (SEH). This is an uncommon condition which can have variable presentations including hemiparesis mimicking stroke, resulting in the potential for misdiagnosis and inappropriate treatment.

## Case presentation

A 28-year-old Chinese male with no past medical history first presented to the emergency department (ED) with sudden onset neck pain radiating to both shoulders and the right lower limb. He had a pain score of 10 out of 10. This was associated with numbness over bilateral upper limbs and the right lower limb. He stated that he does regular weightlifting at the gym and worked as a cabin crew though he had not flown in the preceding year due to the COVID-19 pandemic. He was not known to be taking any supplements or blood thinners. There was no preceding trauma. He had no fever or photophobia. He had no headache, giddiness, nausea, or vomiting. There was also no urinary or bowel incontinence.

On physical examination, the patient was ambulant. He localized his pain to the paravertebral region and bilateral shoulders with no midline spinal tenderness. His sensation and power were full on all four limbs. Cranial nerve and cerebellar examination were normal. Lhermitte’s sign was positive with radiating pain to the right upper limb. Hoffmann’s was negative. The patient declined a digital rectal examination.

X-ray cervical spine was normal. Parenteral analgesia was administered with relief of pain, and he was subsequently discharged.

He re-attended 2 days later in view of neck pain and this was associated with dyspnea and generalized chest pain. He had fallen onto his buttocks when standing up due to right-sided upper and lower limb weakness, which started 9 h before he presented to the ED. His symptom of left upper limb numbness had improved since the first ED visit. He remained dually continent and there was still no headache, vision changes or speech problems. He reported pain over the right upper and lower limbs with a pain score of 8.

On physical examination, there was tenderness over the lower cervical and upper thoracic region. On the Medical Research Council’s (MRC) scale of 0 to 5, his power was 5 for the left upper and lower limbs, and 4 for the right upper and lower limb. Digital rectal examination revealed no abnormalities.

In view of his neurological symptoms coupled with the history of chest pain and dyspnea, he underwent a computed tomography scan of the brain and aortogram. These were reported as normal, with no large ischemic stroke or intracranial hemorrhage or aortic dissection noted.

Seven hours into the ED visit, he reported resolution of his neck pain with some improvement in the power of his right upper limb but also noted deterioration of the power of the right lower limb. He was reviewed by the neurosurgery team in the ED, and their reassessment revealed power 3 for right C5, C7 and 4 for right C6, C8 and T1. The power was 2 over the right lower limb. The power was 5 over the left upper and lower limb. Sensation and proprioception were preserved on all four limbs. There was subtle hyper-reflexia in all four limbs. Plantar reflexes were down going bilaterally. Pulses were well felt in all four limbs. Heart, lungs and abdominal examination remained normal.

The neurosurgery team suggested for the patient to be admitted under the acute stroke unit in view of right hemiparesis, with plans for magnetic resonance imaging of the brain and spine.

### Progress inpatient

His power improved to 4 over his right upper limb and right lower limb by the time he was admitted to the general ward, about 24 h from the time of onset of symptoms.

**Fig. 1:**
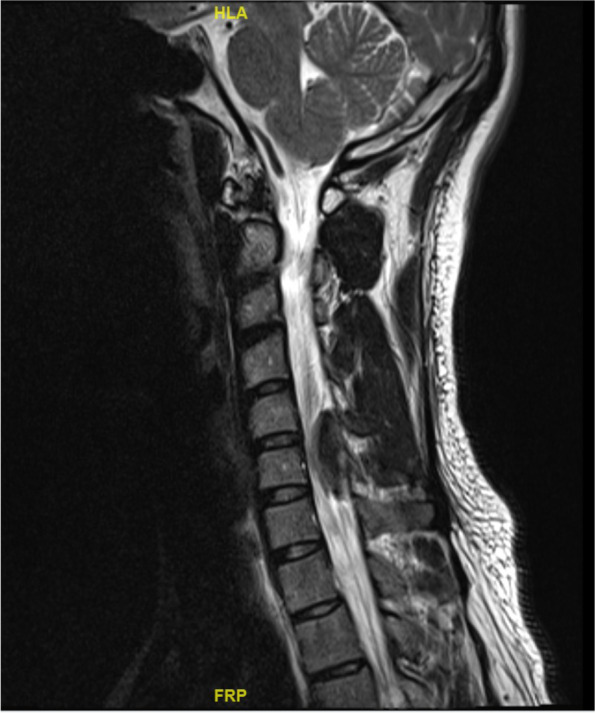
MR cervical spine, T2 sagittal

**Fig. 2:**
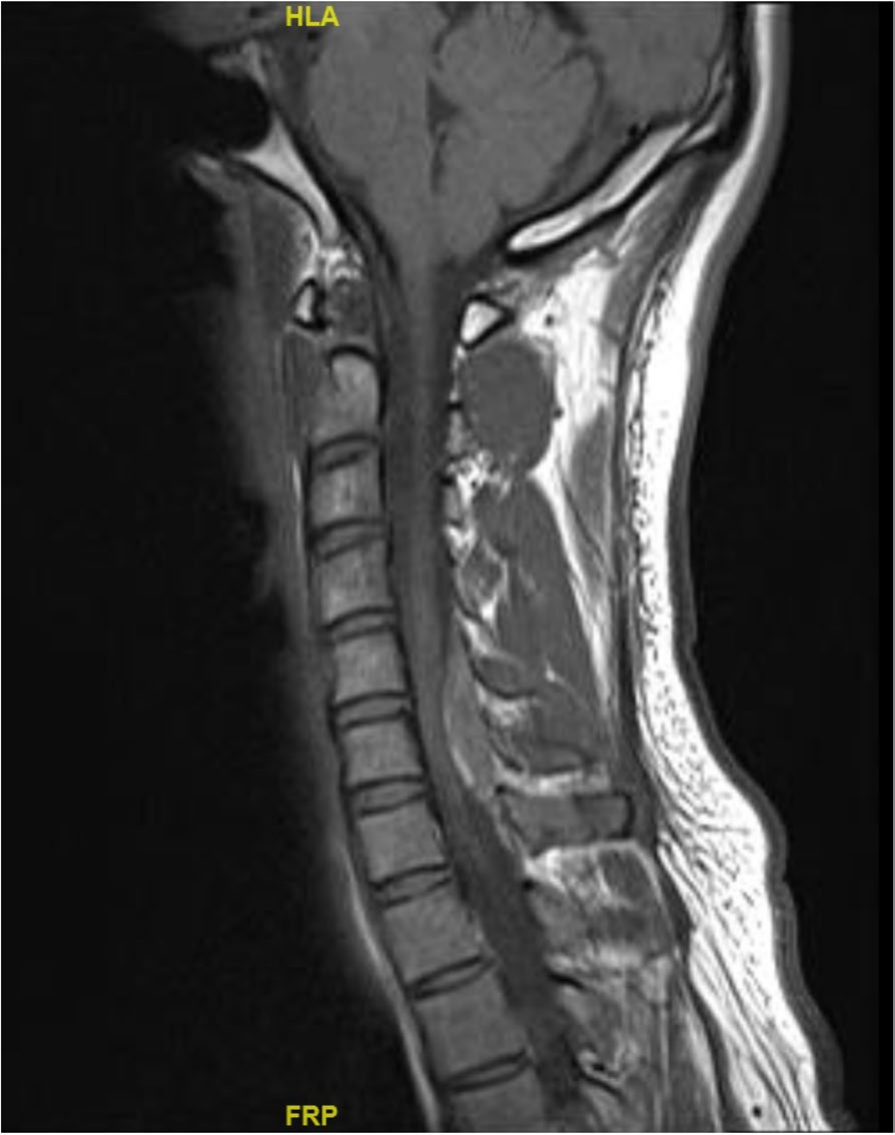
MR cervical spine, T1 sagittal

Magnetic resonance imaging of the brain and whole spine was performed inpatient on the next day (Figs. 1 and 2).

Magnetic resonance imaging of the brain showed no acute infarct or intracranial haemorrhage. Magnetic resonance imaging of the whole spine showed a solitary 3 × 1.9 × 0.8 cm extramedullary, extradural lenticular lesion occupying the C5 to C6 spinal canal posteriorly resulting in significant compression of the underlying cervical cord. The non-enhancing lesion showed T1-hyperintensities and susceptibility, and subtle T2-hyperintensities. Bony alignment was preserved and there was no disc bulge or herniation. The rest of the thoracic and lumbar spine were unremarkable. Overall findings were consistent with an acute cervical spinal epidural hematoma, likely spontaneous.

The patient’s care was taken over by the neurosurgery team. He was counselled for early surgery for spinal cord decompression. However, in view of the significant improvement of his neurological status since initial presentation, the patient was not keen for surgery. He was conservatively managed and had full resolution of right-sided weakness to power 5 on day 3 of admission and resolution of numbness on day 5 of admission. He was also given weaning doses of intravenous to oral dexamethasone. A repeat magnetic resonance imaging of the cervical spine 14 days later showed significant interval decrease in size of the spontaneous posterior epidural hematoma at C5 to C6 (now measuring 2.3 × 0.2 cm), with no spinal canal narrowing or cord compression and no new hematoma.

The patient was advised to undergo spinal digital subtraction angiography (DSA) to evaluate for possible arterio-venous malformation, but he declined, as he was not keen for an invasive procedure.

He also developed unexplained bruising over his limbs on day 8 of admission. Haematological workup for bleeding disorders including platelet, coagulation profile, factor assays, von Willebrand and myeloma panel were unremarkable.

The patient was discharged on day 14 of admission and was reviewed in the neurosurgery clinic 3 weeks after discharge. He remained well with power 5 over all four limbs.

## Discussion and conclusions

Occurrence of spontaneous spinal epidural haematoma (SEH) in the cervical region can present with hemiplegia without facial involvement. SEH is an important differential diagnosis to consider in patients presenting with stroke-like symptoms. A misdiagnosis as cerebral infarction may lead to mistreatment with administration of thrombolytics if presenting within the thrombolytic window or anti-platelets which would worsen the hematoma, resulting in worsening neurological symptoms [1]. On the other hand, SEH can also be easily dismissed as musculoskeletal pain in young patients; hence, it is important for the clinician to obtain a detailed history and perform a thorough physical examination to avoid missing this diagnosis.

Though stroke is a common cause of hemiparesis, it is not usually associated with pain. Sudden onset neck or back pain, radiating to the limb or trunk, is a common presenting complaint [2] in patients with spontaneous SEH and this should prompt the clinician to consider SEH in patients with acute hemiparesis. Paraparesis and tetraparesis are more common presentations of SEH than hemiparesis [3]. Other differentials of acute face sparing hemiparesis with cervicothoracic pain includes compressive lesions such as tumours and herniated disc of the cervical spine, carotid artery and vertebral artery dissection. Proper imaging coupled with history and physical examination will allow one to reach the right diagnosis.

The general incidence of spontaneous SEH is estimated to be 0.1 patients per 100,000 patients per year [4]. It is more common than spontaneous spinal subdural hematoma due to the relatively larger and more vascular epidural space occupied by loose fatty tissue and Batson’s vertebral plexus [5]. Domenicucci et al. did a literature review of 959 cases and the mean patient age was 48 years (range 0–91 years), and 60% of patients were male. A bimodal distribution has been reported for age at onset with peaks in the second and sixth decades of life. The most common sites for SEH were C6 and T12, with maximum extension of six vertebral bodies in 75% [6].

Pathogenesis of SEH is not completely understood. Domenicucci et al. analysed 1010 cases in which the cause of the SEH was not reported in 42% of cases. The aetiology ranged from iatrogenic factors (18%), such as drug-induced coagulopathy or spinal puncture, to non-iatrogenic factors (29%), such as genetic or metabolic coagulopathy, trauma and pregnancy. The aetiology was multifactorial in 11.1% of cases [6]. There have also been reports of vascular malformations contributing to the development of SEH [7]. Some believe that the internal posterior epidural venous plexus is the anatomic structure responsible for the hematoma [8]. Causes which rapidly increase the intra-abdominal and thoracic pressure, such as pregnancy, coughing, sneezing, vomiting and urinating, may cause venous epidural plexus lesions [9]. When it comes to minor trauma, it may arise from significant dynamic stress, such as stretching exercises, injuries during physical activities, sports, daily routine activities, chiropractic spinal manipulation therapy or acupuncture [10–12]. There is also association of SEH with rheumatological conditions such as ankylosing spondylitis, but usually in a traumatic setting [13, 14]. Our patient participated in regular weightlifting. Sporting weightlifting or lifting heavy objects in young healthy subjects has been identified as a risk factor for SEH [15–18].

**Fig. 3:**
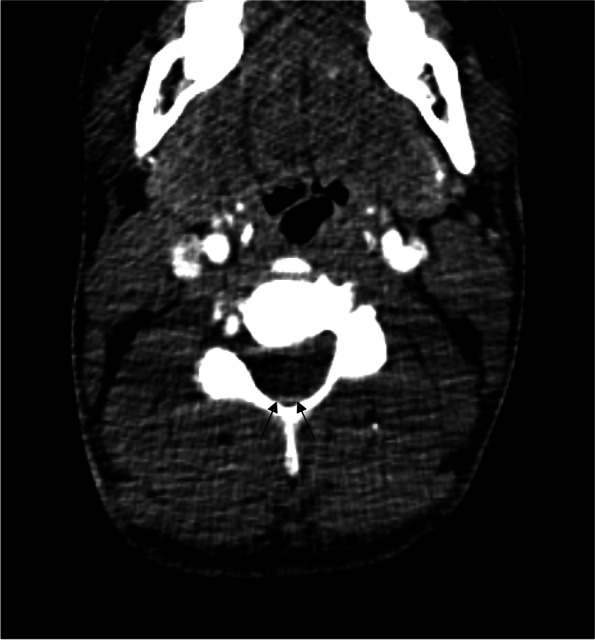
CT aortogram, arterial phase, C5 level. Arrows pointing to subtle hyperdensity

Magnetic resonance imaging (MRI) is the gold standard, but it may not be the most practical modality in the ED. Computed tomography of the cervical spine is readily available and has been shown to pick up SEH which is seen as a subtle hyper density in the spinal canal that can be easily missed without clinical suspicion [7, 19]. CT imaging also has the added advantage of concurrently assessing for other aetiologies such as stroke and aortic dissection which can present with similar symptoms. In our patient, the presentation of hemiparesis and chest pain leads to the prioritization of CT brain and aortogram in the emergency setting to rule out a stroke or an aortic dissection instead of a CT cervical angiogram. A retrospective review of the CT aortogram images revealed a subtle hyper density in the spinal canal at the C5 level (Fig. 3). Hence, if the clinical index of suspicion of SEH is high, a CT can be a useful imaging tool if MRI is not readily available. It has also been suggested that CT angiogram can be utilized to rapidly monitor patients who have worsening pathology. However, the clinician must bear in mind that the CT findings may be subtle and smaller hematomas can be missed [20].

Fortunately, the patient had spontaneous resolution of neurological symptoms with conservative management. There is a lack of consensus on the decision between surgical and conservative management. Surgical management is considered the mainstay of treatment for SEH. It is critical to intervene early surgically as a recent meta-analysis showed that the duration of paralysis pre-operatively is the only independent predictor of poor outcome [21].

Conservative management is only recommended for patients with minimal neurological deficits, improvement of symptoms over short period of time and imaging studies showing dissipation of SEH [22]. Spontaneous resolution of SEH usually occurs several hours to days after onset [23]. This has been hypothesized to be due to the spreading of the hematoma along the spinal epidural space though no conclusive evidence has been reached [24, 25]. If there is a lack of neurological improvement or any sign of deterioration, the patient should be emergently treated with surgery [26].

In conclusion, SEH, although uncommon, can be a mimic of stroke and it is important to avoid misdiagnosis as it is a time critical diagnosis, and administration of thrombolysis or antiplatelets can lead to unfavourable outcomes. Having a high clinical suspicion can help to guide us in the choice of imaging and interpretation of subtle signs to reach the correct diagnosis in a timely manner. Further research is required to better understand the factors that would favour a conservative approach as opposed to surgical treatment.

## Data Availability

Not applicable

## References

[1] Watanabe M, Abe E, Sakamoto K (2020). Analysis of a spontaneous spinal epidural hematoma mimicking cerebral infarction: a case report and review of the literatures. No Shinkei Geka.

[2] Thiele RH, Hage ZA, Surdell DL (2008). Spontaneous spinal epidural hematoma of unknown etiology: case report and literature review. Neurocrit Care.

[3] Hsieh CF, Lin HJ, Chen KT (2006). Acute spontaneous cervical spinal epidural hematoma with hemiparesis as the initial presentation. Eur J Emerg Med.

[4] Holtås S, Heiling M, Lönntoft M (1996). Spontaneous spinal epidural hematoma: findings at MR imaging and clinical correlation. Radiology..

[5] Vinters HV, Barnett HJ, Kaufmann JC (1980). Subdural hematoma of the spinal cord and widespread subarachnoid hemorrhage complicating anticoagulant therapy. Stroke..

[6] Domenicucci M, Mancarella C, Santoro G (2017). Spinal epidural hematomas: personal experience and literature review of more than 1000 cases. J Neurosurg Spine.

[7] Zhong W, Chen H, You C, Li J, Liu Y, Huang S (2011). Spontaneous spinal epidural hematoma. J Clin Neurosci.

[8] Groen RJ (2004). Non-operative treatment of spontaneous spinal epidural hematomas: a review of the literature and a comparison with operative cases. Acta Neurochir.

[9] Hsieh CT, Chiang YH, Tang CT (2007). Delayed traumatic thoracic spinal epidural hematoma: a case report and literature review. Am J Emerg Med.

[10] Cooper J, Battaglia P, Reiter T (2019). Spinal epidural hematoma in a patient on chronic anticoagulation therapy performing self-neck manipulation: a case report. Chiropr Man Therap.

[11] Lidder S, Lang KJ, Masterson S, Blagg S (2010). Acute spinal epidural haematoma causing cord compression after chiropractic neck manipulation: an under-recognised serious hazard?. J R Army Med Corps.

[12] Chen CL, Chang MH, Lee WJ (2020). A case report: an acute spinal epidural hematoma after acupuncture mimicking stroke. J Emerg Med.

[13] Tamburrelli FC, Meluzio MC, Masci G (2018). Etiopathogenesis of traumatic spinal epidural hematoma. Neurospine..

[14] Anipindi S, Ibrahim N (2017). Epidural haematoma causing paraplegia in a patient with ankylosing spondylitis: a case report. Anesth Pain Med.

[15] Latzka E, Neelwant SN (2016). Acute radiating low back pain after olympic-lifting. Clin J Sport Med.

[16] Vitali AM, Steinbok P (2008). Spontaneous spinal epidural hematoma following weight lifting. Can J Neurol Sci.

[17] Chen CJ, Fang W, Chen CM, Wan YL (1997). Spontaneous spinal epidural haematomas with repeated remission and relapse. Neuroradiology..

[18] Kingery WS, Seibel M, Date ES, Marks MP (1994). The natural resolution of a lumbar spontaneous epidural hematoma and associated radiculopathy. Spine (Phila Pa 1976).

[19] Lim SW, Wong E (2020). Spontaneous epidural hematoma of the cervical spine in an elderly woman with recent COVID-19 infection: a case report. Am J Case Rep.

[20] Huang D, Iken S, Elbadri S, Falgiani M, Ganti L (2022). Spontaneous spinal epidural hematoma: a case of a benign presentation and emergency department management. Cureus..

[21] Bakker NA, Veeger NJ, Vergeer RA, Groen RJ (2015). Prognosis after spinal cord and cauda compression in spontaneous spinal epidural hematomas. Neurology..

[22] Zhang S, Geng F, Wang J (2018). Rapid recovery of spontaneous spinal epidural hematoma without surgical treatment: case report and literature review. World Neurosurg.

[23] Huh J, Kwak HY, Chung YN (2016). Acute cervical spontaneous spinal epidural hematoma presenting with minimal neurological deficits: a case report. Anesth Pain Med.

[24] Serizawa Y, Ohshiro K, Tanaka K, Tamaki S, Matsuura K, Uchihara T (1995). Spontaneous resolution of an acute spontaneous spinal epidural hematoma without neurological deficits. Intern Med.

[25] Hentschel SJ, Woolfenden AR, Fairholm DJ (2001). Resolution of spontaneous spinal epidural hematoma without surgery: report of two cases. Spine (Phila Pa 1976).

[26] Raasck K, Habis AA, Aoude A (2017). Spontaneous spinal epidural hematoma management: a case series and literature review. Spinal Cord Ser Cases.

